# Circulating Non-Coding RNAs in Head and Neck Cancer: Roles in Diagnosis, Prognosis, and Therapy Monitoring

**DOI:** 10.3390/cells10010048

**Published:** 2020-12-31

**Authors:** Araceli Diez-Fraile, Joke De Ceulaer, Charlotte Derpoorter, Christophe Spaas, Tom De Backer, Philippe Lamoral, Johan Abeloos, Tim Lammens

**Affiliations:** 1 Division of Oral and Maxillofacial Surgery, Department of Surgery, AZ Sint-Jan Brugge-Oostende A.V., 8000 Bruges, Belgium; araceli.diez-fraile@azsintjan.be (A.D.-F.); joke.deceulaer@azsintjan.be (J.D.C.); christophe.spaas@azsintjan.be (C.S.); tom.debacker@azsintjan.be (T.D.B.); philippe.lamoral@azsintjan.be (P.L.); johan.abeloos@azsintjan.be (J.A.); 2 Department of Pediatric Hematology-Oncology and Stem Cell Transplantation, Ghent University Hospital, 9000 Ghent, Belgium; charlotte.derpoorter@ugent.be; 3 Department of Internal Medicine and Pediatrics, Ghent University, 9000 Ghent, Belgium; 4 Cancer Research Institute Ghent (C.R.I.G.), 9000 Ghent, Belgium

**Keywords:** head and neck cancer, circulating non-coding RNA, liquid biopsy, biomarkers

## Abstract

Head and neck cancer (HNC), the seventh most common form of cancer worldwide, is a group of epithelial malignancies affecting sites in the upper aerodigestive tract. The 5-year overall survival for patients with HNC has stayed around 40–50% for decades, with mortality being attributable mainly to late diagnosis and recurrence. Recently, non-coding RNAs, including tRNA halves, YRNA fragments, microRNAs (miRNAs), and long non-coding RNAs (lncRNAs), have been identified in the blood and saliva of patients diagnosed with HNC. These observations have recently fueled the study of their potential use in early detection, diagnosis, and risk assessment. The present review focuses on recent insights and the potential impact that circulating non-coding RNA evaluation may have on clinical decision-making in the management of HNC.

## 1. Introduction

**Figure 1:**
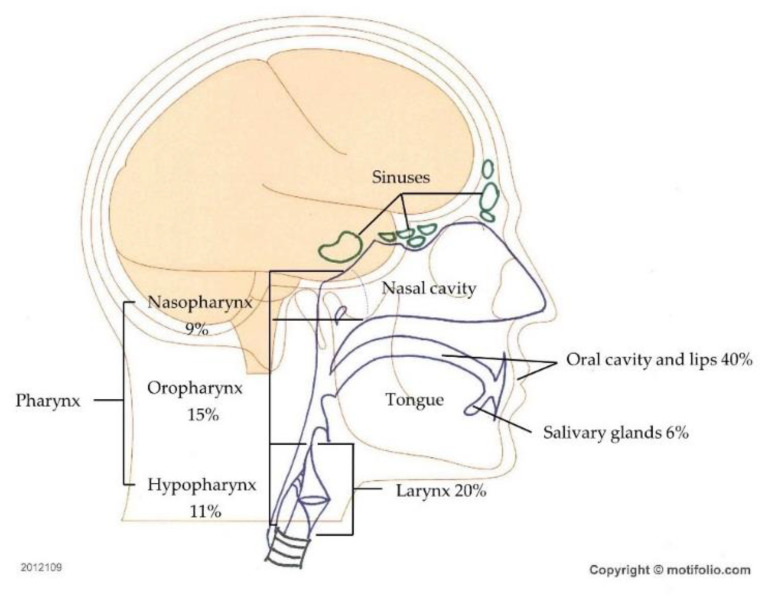
Head and neck cancer (HNC) types. Regions and frequencies where head and neck cancers are diagnosed are indicated.

Head and neck cancer (HNC) encompasses a wide range of tumors arising from numerous anatomic subsites, including the lips, oral cavity, oropharynx, nasopharynx, hypopharynx, larynx, nasal cavity, paranasal sinuses, and salivary glands (Figure 1). Squamous cell carcinoma (SCC) is the most frequent histological type, accounting for 90% of HNC cases [1]. Globally, HNC the seventh most frequent malignancy, with more than 880,000 new cases reported in 2018, representing 4.9% of the total number of cancer cases [2]. HNC has a high incidence and mortality profile worldwide, with age-standardized incidence and mortality rates of 10.10 and 5.04 per 100,000, respectively, in 2018 [2]. In western Europe, Jethwa et al. [3] found that males were affected three times as frequently as females, with the highest rates of HNC occurring in older men, and found that the risk factors for HNC included upper aerodigestive tract mucosa exposure to carcinogens such as tobacco and alcohol [3]. While smoking-related cancers appear to be declining, there have been increasing incidences of SCCs of the oropharynx and nasopharynx in young individuals in recent years; these increases have been linked to infection with human papillomavirus (HPV) and, less commonly, Epstein–Barr (EBV) virus [1,4]. The observation of substantially better prognoses for these malignancies among HPV positive patients than for tobacco users has suggested that activation of HPV-specific oncogenic signaling pathways may be relevant for HNC prognosis [5,6,7,8].

Typically, HNCs present with symptoms from the primary site, such as persistent hoarseness, long-lasting dysphagia, oral mucosa ulcers, epistaxis, or otalgia. Patients in whom the primary neoplastic site is the tongue base, upper glottis, or nasopharynx often present with cervical lymphadenopathy as their first presenting sign. Most patients (60%) are diagnosed at an advanced stage of disease (III or IV), at which points survival rates are reduced relative to those for early stages (I or II). Distant metastases are found in only about 10% of cases at presentation, and concomitant or delayed second primary tumors of the upper aerodigestive tract occur in 10–15% of patients. Despite recent advances in loco-regional treatment protocols, including advanced surgery, radiotherapy, chemotherapy, or combinations thereof, 50–60% of patients with locally advanced disease develop loco-regional recurrence and a further 20% progress to distant metastasis [9]. Challenges in HNC care include rapid detection of primary tumors during the early stages of the disease, the development of improved surveillance methods following potentially curative treatment, unambiguous distinction of metastasis from recurrences and second primary tumors, and the development of therapeutic options for cases that are currently considered untreatable.

In response to these difficulties, new screening modalities have emerged in multiple areas of oncology. Key among these are liquid biopsies, wherein tumor cells or tumor-derived products released into body fluid are collected and examined with the aim of detecting cancer biomarkers [10]. Multiple biofluids can be used for analysis, with peripheral blood being the most commonly investigated specimen. Compared to classical excisional biopsies, blood sampling is less invasive and it holds the promise of rapid, safe, and highly informative genetic analysis. Hence, blood sampling provides an accessible means of real-time evaluation of the disease and, potentially, the opportunity to identify small tumors not yet visible by medical imaging techniques. It is important to bear in mind that the main costs of a liquid biopsy are the associated laboratory studies and downstream data analysis. Ideally, liquid biopsies should be inexpensive enough to be analyzed at multiple time points during treatment. Such repeat monitoring can improve HNC outcomes while reducing patient morbidity due to unnecessary treatments, boosting treatment development and optimizing the use of healthcare resources [11].

Various types of circulating tumor-derived products have been identified, including circulating tumor cells (CTCs) as well as biomolecules including circulating tumor DNA (ctDNA) and circulating tumor RNA (ctRNA) [10]. Isolation, detection, and characterization of CTCs can provide crucial information regarding disease stratification at the point of diagnosis based on the mutational status of the tumor, chromosomal abnormalities, methylation status, and the overall tumor heterogeneity and complexity [12]. The clinical use of CTCs has been hindered by detection limits due to low presence in early disease stages and due to heterogeneity of metastatic potential. Biomolecules such as ctDNA and ctRNA are highly promising biomarker candidates. Clonal events can be investigated through ctDNA studies and CTC studies. Conversely, compared with DNA biomarkers, RNA biomarkers provide better dynamic insights into cell regulation and states. In most body fluids, ncRNA stability is warranted through diverse transport mechanisms including encapsulation into membranous vesicles such as exosomes, microvesicles, apoptotic bodies, and association to RNA-binding proteins, or lipoprotein complexes [13,14,15,16,17,18]. In addition, tRNAs and tRNA-derived fragments are known to possess nucleotide modifications at multiple sites, which bear important roles for the stabilization of tRNAs [19]. However, since these molecules are affected by biological processes throughout the body, other concurrent biological events can have confounding effects. For example, DNA released from apoptotic leukocytes is a major source of contaminants, especially when taking into account post-chemotherapeutic-associated non-targeted cellular death.

The biomarker potential of ctRNA analytes has been explored in recent years and several research groups have provided consolidating evidence of ctRNA implications in oncogenic processes and of their use as biomarkers in HNC [20,21]. Evidence is accumulating rapidly in this emerging field. In this review, we will provide an overview of ctRNA types and an update regarding research progress related to the biological and clinical significance of non-coding ctRNA types as biomarkers for HNC diagnosis, risk assessment, and monitoring.

## 2. CtRNA: Types, Biogenesis, and Roles

**Figure 2:**
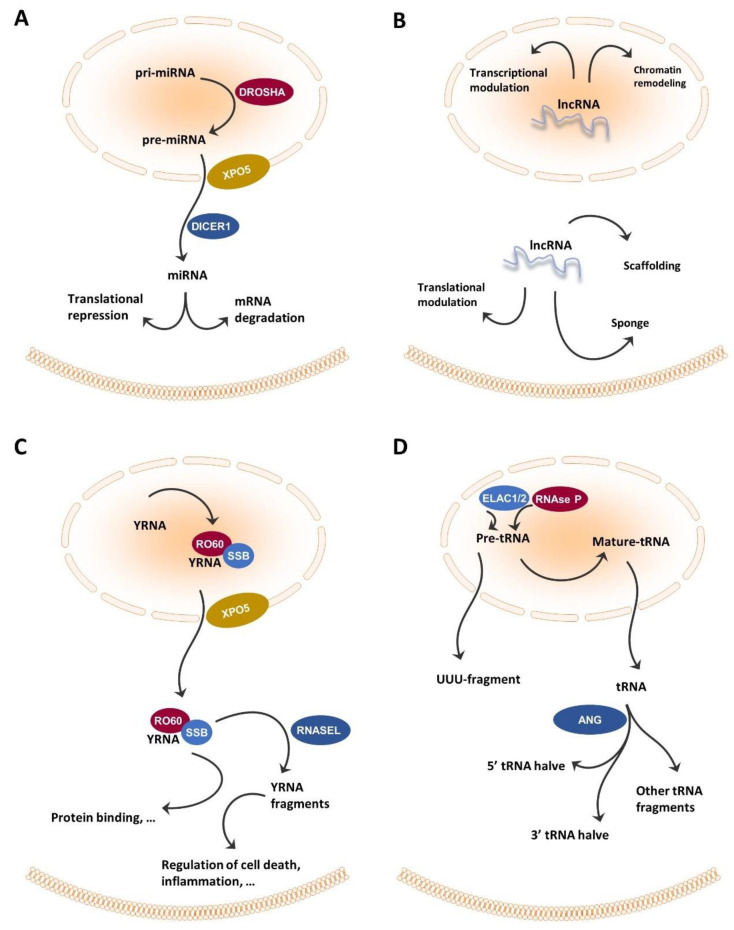
Biogenesis and functions of non-coding RNAs found in the circulating system in HNC patients. (**A**) Pri-miRNAs are converted into pre-miRNA by DROSHA, exported from the nucleus to the cytoplasm by XPO5, cleaved by DICER1, and assembled into the RISC complex, which allows translational repression or mRNA degradation. (**B**) lncRNAs engage in many cellular functions including miRNA sponging, protein scavenging, acting as a scaffold for proteins functioning together, chromatin remodeling support, and contributing active (or silencing) gene transcription and modulating mRNA functions. (**C**) ScnRNA, called YRNA, is transcribed (chromosomal location 7q36) and bound by SSB and RO60 proteins. In the cytoplasm YRNA is broken into fragments. (**D**) tRNAs are transcribed in the nucleus via RNA polymerase III as large precursors with 5′-leader and 3′-trailer sequences. The 5′-leader sequence is cleaved out by RNase P and the 3′-trailer sequence is removed by ELAC1/2 (a.k.a. RNase Z) to yield mature tRNAs. tRNA halves are generated by anticodon-cleaving enzyme ANG.

Some 60–70% of the transcriptome consists of non-coding RNA. After long being ignored, non-coding RNA has since been shown to be involved in normal development and disease [22,23]. Hombach and Kretz [24] suggested distinguishing small non-coding RNAs (sncRNAs), with 200 nucleotides (nt) from long non-coding RNAs (lncRNAs), with 200 nt; circular RNAs are included in the latter group (Figure 2). In the following paragraphs, currently used non-coding RNA classes are described, with an emphasis on those classes that are germane to the current review.

### 2.1. miRNA

miRNAs are a highly conserved class of RNAs accounting for approximately 1% of the total number of estimated RNA molecules in cells [25]. They occur in intergenic, intronic, and exonic regions as hairpin-shaped precursors, and their expression involves the transcriptional machinery of protein-coding genes, including the participation of RNA polymerase II. Further processing into mature miRNAs is performed by the Drosha and Dicer enzymes [26,27]. Mature miRNAs are 19~25 nt long and bind specific sites in 3′ untranslated regions of target mRNAs. This process can suppress translation or target degradation [26,27,28,29]. Importantly, individual miRNAs can have either oncogenic or tumor-suppressive functions. Although the majority of miRNAs are reported to be involved in general oncogenic pathways (e.g., let-7, miR-21, miR-155), it was demonstrated that miRNA profiles reflect the developmental lineage of tumors and are therefore able to accurately distinguish between multiple tumor types [30,31].

### 2.2. tRNA and tRNA-Derived Fragments

In recent years, an increasing number of studies have found that the RNA polymerase III-transcribed pre- or mature tRNAs are cleaved into tRNA-derived small RNAs (tsRNAs), tRNA-derived fragments (transfer RNA-derived RNA fragments, tRFs), and tRNA halves known as tRNA-derived stress-induced small RNAs (tiRNAs) [32]. Our understanding of tRFs and tiRNAs is improving at a fast pace, and multiple research teams have shown that they have various biological functions including acting as miRNAs to regulate translation, gene expression, and cellular stress responses [33,34]. Decades ago scientists had already pinpointed the excretion of tRNA fragments in urine of different cancer patients, varying in level with stage of the disease [35]. Recently, tRNA-derived fragments have been shown to be involved in human disease and, more specifically, in cancer cell proliferation, metastasis, progression, and survival [36,37,38].

### 2.3. YRNA and YRNA-Derived Small RNAs

YRNAs and YRNA-derived small RNAs were discovered only recently. Human YRNA genes are clustered on a single chromosomal locus at chromosome 7q36. Four YRNA transcripts have been identified: YRNA1 (112 nt, 35.7 kDa), YRNA3 (101 nt, 32.2 kDa), YRNA4 (93 nt, 30.0 kDa), and YRNA5 (83 nt, 27.6 kDa) [27]. Although it would be expected based on their stem-loop structure, YRNAs do not undergo miRNA-like biological processing. Instead, they are transcribed in the nucleus by RNA polymerase III, and bound by SSB protein for nuclear retention or bound to RO60 to facilitate nuclear export [39]. YRNA degradation by caspase-3 in apoptotic cells generates YRNA-derived fragments, with one type being 22~25 nt and the other being 27~36 nt [39]. Additionally, RNAse L produces YRNA-derived small RNAs in response to ultraviolet light exposure [40]. Research into YRNA and YRNA-derived small RNAs is emergent. Following their identification as an essential factor for chromosomal DNA replication in human cell nuclei, it was shown that YRNAs are overexpressed in solid tumors compared to normal tissue counterparts [41,42]. Interestingly, a tumor-type specific pattern of overexpression was reported [41,42]. Recent studies have shown that the expression patterns of YNRA and YRNA-derived small RNAs are altered in several diseases, including coronary artery disease, Sjogren syndrome, and cancer [40,43].

### 2.4. lncRNA

lncRNAs are RNA transcripts that are longer than 200 nt, transcribed by RNA polymerase II, and lacking coding potential due to being devoid of evident open reading frames [44]. Different initiatives have led to the identification, categorization, and annotation of tens of thousands of lncRNAs, which are listed in public databases such as LNCipedia and lncRNAdb [45,46]. Current thinking implies that lncRNAs function by forming complexes with protein and RNA molecules inside and outside of the nucleus. lncRNAs are often poly-adenylated and, compared with coding genes, are usually less well conserved, more tissue-specific, and less abundant [24,47]. In addition, their expression has been reported to be tissue- and disease-specific, exemplified by the neuroblastoma- and melanoma-specific expression of lncNB1 and SAMMSON, respectively [48,49]. Despite ongoing research, the functions of the vast majority of lncRNAs are still unknown. However, lncRNA function broadly falls into two categories: cis regulators of local chromatin structure and/or gene expression, and trans mediators of cellular functions distant from their sites of transcription [50]. Recently, lncRNAs have been implicated as key regulators in various biological processes, including the pathogenesis and progression of cancer [47,51]. lncRNAs can exert both tumor-suppressive and oncogenic effects and thus could be useful as biomarkers or, potentially, as therapeutic targets.

## 3. Impact on Diagnosis

### 3.1. Role of miRNAs

**Figure 3:**
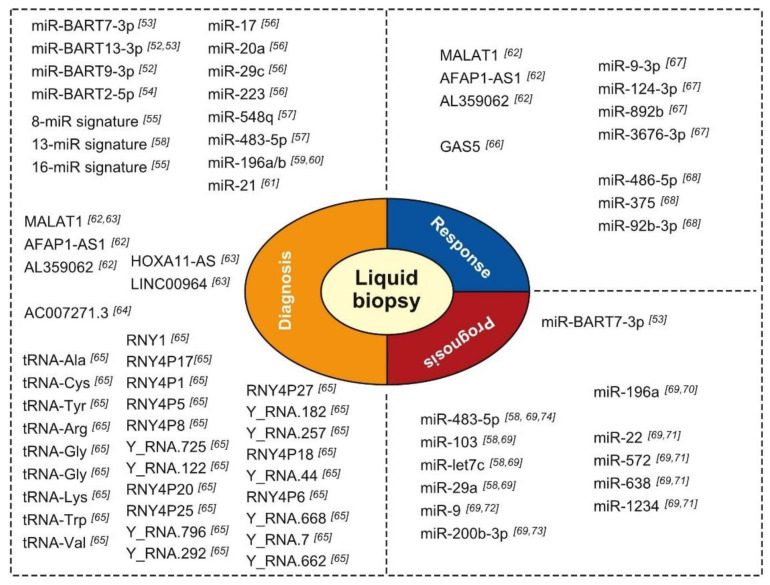
Condensed overview of circulating non-coding RNAs with purported diagnostic, prognostic, or treatment response prediction value in HNC [52,53,54,55,56,57,58,59,60,61,62,63,64,65,66,67,68,69,70,71,72,73,74].

Not unsurprisingly, the expression of miRNAs in blood has been a major focus of investigation into the use of non-coding RNA expression in HNC diagnosis. Considering the role of EBV infection in the etiology of nasopharyngeal cancer (NPC), several research teams investigated the presence of circulating EBV-related miRNAs in NPC patients (Figure 3). Interestingly, at least two independent teams showed that the miRNAs BART7-3p and miR-BART13-3p can be useful in the diagnosis of early and late-stage NPC with high sensitivity and specificity [52,53]. Detection of these miRNAs surpassed the performance of EBV-DNA detection and immune-based assays in NPC diagnosis. Additionally, preliminary evidence suggests that BART9-3p and BART2-5p may be useful diagnostic markers in NPC. In a study with a cohort of high-risk patients conducted as part of a screening program, expression of the miRNA BART2-5p enabled detection of NPC prior to the development of clinical signs, suggesting that it may be used as a preclinical marker, with an area under the received operating characteristic curve (AUC) of 0.858 and 95% confidence interval (CI) of 0.765–0.951. There are no serological tests for preclinical markers of NPC to date [54].

In addition to evaluating EBV-miRNA expression, Wen and colleagues [55] have undertaken a broad microarray-based screening project using whole blood from cancer-free controls and patients with different HNC subtype diagnoses. They distilled an 8-miRNA signature that distinguishes patients with NPC from healthy controls (HCs) with a 97.14% accuracy (sensitivity, 96.43%; specificity, 100%) and an AUC of 0.995 (*p* 0.001). These results were validated in an independent cohort with an accuracy of 86.67% (sensitivity, 86.11%; specificity, 88.89%). Similarly, a 16-miRNA signature differentiating NPC from other HNCs was identified. This latter model provided 100% accuracy, sensitivity, and specificity in a training cohort, and 94.44% sensitivity and 72.22% specificity in a validation cohort. Other studies examining the utility of serum miRNA expression in NPC patients have pointed to the diagnostic utility of expression analysis of miRNA combinations involving miR-17, miR-20a, miR-29c, miR-223, miR-548q, miR-483-5p, miR-103, miR-29a, and miR-31-5p [56,57,58]. The muddle of putative signatures suggested (see Table 1), however, awaits thorough comparative evaluations to determine the most performant combination.

Lu and colleagues [59,60] undertook yet another approach to identifying oral cancer diagnostic miRNAs. They identified miR-196a/b as being highly significantly differentially expressed in oral cancer lesions compared to normal keratinocytes. Interestingly, in a subsequent study, they showed that expression of both miR-196a and miR-196b can discriminate among samples from healthy donors (*n* = 50), precancerous lesions (*n* = 16), and oral cancer tumors (*n* = 90) with the following AUCs: miR-196a oral cancer vs. healthy sample, AUC = 0.864; miR-196a precancerous vs. healthy sample, AUC = 0.760; miR-196b oral cancer vs. healthy sample, AUC = 0.960; and miR-196b precancerous vs. healthy sample, AUC = 0.840 [60]. Finally, in their recent meta-analysis review of seven original research reports, Dioguardi and colleagues [61] found strong support for the oral cancer diagnostic value of miR-21 expression in serum/plasma, with an aggregated odds ratio of 7.620 (95% CI, 3.613–16.070), aggregated sensitivity of 0.771 (95% CI, 0.680–0.842) (*p* 0.001), and an aggregate specificity of 0.663 (95% CI, 0.538–0.770) (*p* 0.001).

We found only one study that included a thorough investigation of saliva-borne non-coding RNAs in NPC diagnosis. Using a microarray containing probes for 2025 miRNAs, Wu and colleagues [75] found that NPC saliva can be distinguished from HC saliva based on the expression of 4 upregulated and 47 downregulated miRNAs. Furthermore, they found that the expression of a set of 12 of these downregulated miRNAs provided excellent discrimination of NPC patients from HCs (AUC = 0.999; 95% CI, 0.923–1.000) with 100.0% sensitivity and 96.0% specificity [75]. Interestingly, several of the abnormally regulated miRNAs were predicted to target endocytosis-related genes, and thus might play a role in EBV cell entry [75].

### 3.2. Role of lncRNAs

In sharp contrast to the massive volume of data published on circulating miRNA expression for diagnosing HNCs, only a few researchers have tackled the expression of circulating lncRNAs. In an elegantly designed study, He and colleagues [62] combined expression of lncRNAs in cell lines, 101 NPCs, 20 patients with chronic nasopharyngitis (CN), 20 EBV carriers (ECs), 101 HCs. They showed that NPC detection can be done based on serum levels of MALAT1, AFAP1-AS1, AL359062, with AUCs of 0.918 for NPC vs. HC, 0.893 for NPC vs. CN, and 0.877 for NPC vs. EC. Moreover, the expression of these three lncRNAs discriminated even early-stage NPC patients (T1-2) from HCs (AUC = 0.833), CN patients (AUC = 0.824), and EC patients (AUC = 0.800). These data support the notion that lncRNAs may have important diagnostic potential. Furthermore, knock-down of the lncRNA AL359062 in NPC cell lines resulted in reduced migration, invasiveness, and proliferation, indicating that this lncRNA, and perhaps others, could be therapeutic targets [62]. Pursuing an unbiased approach, Yao and colleagues [63] screened plasma samples using sequencing and microarrays for lncRNAs whose expression in plasma was associated with a diagnosis of head and neck squamous cell carcinoma (HNSCC). Filtering, validation in larger sample sets, and cross-validation of expression in tissue led to the identification of three lncRNAs that were predictive of a diagnosis of HNSCC: HOXA11-AS, LINC00964, and metastasis-associated lung adenocarcinoma transcript 1 (MALAT1). The combined expression of these three lncRNAs into one risk score allowed Yao and colleagues [63] to distinguish patients with HNSCC from HC donors with AUCs of 0.925 and 0.839 in independent discovery and validation cohorts, respectively. Finally, Shao et al. [64] showed that serum expression of lncRNA AC007271.3 combined with serum SCC antigen presence could be used to distinguish oral SCC patients from normal controls with an AUC of 0.902 (0.849–0.955), sensitivity of 82.5%, and specificity of 91.4%. Moreover, they found that AC007271.3 expression was associated with degree of pathological differentiation (*p* = 0.022), clinical stage (*p* = 0.005), and lymphatic metastasis (*p* = 0.011), but not with age, sex, or alcohol history [64].

### 3.3. Role of YRNAs and tRNAs and Derived Fragments

The involvement of YRNAs, tRNAs, and their derivatives in HNCs represent a largely unexplored field of study. Recently, Dhahbi and colleagues [76] performed a small RNA sequencing study of serum samples from patients with oral SCC, focusing on the expression of tRNA halves and YRNAs. Interestingly, they found that the expression of 22 tRNA halves and four YRNA fragments were decreased in the serum of oral SCC patients. However, only one tRNA half and no YRNA fragments could be confirmed to be downregulated in cancerous tissues [76]. In a previous deep sequencing study of serum/plasma sncRNAs, Martinez and colleagues [65] found that, compared to samples from HCs, samples from patients diagnosed with HNSCC had significantly increased circulating levels of 5′ tRNA halves derived from iso-acceptors of tRNAAla, -Cys, and -Tyr, together with decreased circulating levels of 5′ tRNA halves derived from tRNA-Arg, -Glu, -Gly, -Lys, -Trp, and -Val. They identified 19 types of YRNA-derived small RNAs that were decreased in HNSCC samples, including 11 that were derived from the 3′ end and 8 that were derived from the 5′ end of YRNA genes. Only two YRNA-derived sncRNAs were increased in abundance in HNSCC samples, both of which were derived from the 5′ ends of YRNA genes [65].

## 4. Response Evaluation, Residual Disease, and Recurrence

Monitoring of therapeutic response and quantifying residual disease offer opportunities to enhance survival chances. For example, evaluation of minimal residual disease and concurrent risk stratification has led to risk-adapted treatment protocols with increased therapeutic success in patients with childhood acute lymphoblastic leukemia [77,78]. This application has yet to be explored with non-coding RNAs in HNC liquid biopsies. However, Fayda and colleagues [66] found that plasma levels of the lncRNA GAS5 were significantly higher in samples from HNC patients with a partial therapeutic response or progressive disease than in samples from patients who showed a clinical response to therapies. Following up on the reporting of 33 miRNAs with differential expression in NPC samples relative to control samples Xu et al. [67] documented fluctuating miRNA expression levels in plasma from NPC patients at diagnosis and in 3-, 6-, and 12-month post-radiochemotherapy serum samples. Importantly, they found that the dynamic changes of expression levels of four miRNAs during follow-up were predictive of NPC recurrence [67]. Similarly, in a study of oral SCC, Yan et al. [68] found that miR-486-5p, miR-375, and miR-92b-3p expression levels changed postoperatively compared to pre-operatively, and that absence of these changes was associated with oral SCC recurrence 9–12 months after surgery [68]. Similarly, He et al. [62] found that persistence of high serum levels of MALAT1, AFAP1-AS1, and AL359062 lncRNAs following treatment was associated with recurrence, residual NPC in lymph nodes, or metastasis.

## 5. Impact on Prognosis

Studies examining the prognostic value of serum levels of tumor-associated antigens [79], proinflammatory cytokines [80,81], hormones, and other molecules [82] have yielded inconsistent results. Few studies have evaluated the potential of circulating non-coding RNAs as prognostic biomarkers in HNC. In a recent meta-analysis of six high-quality studies (1000 patients; only reports including key statistics such as Hazard Ratio and Confidence Intervals) examining the potential prognostic impact of blood-borne miRNAs, Lamichhane et al. [69] found that poor survival of patients with HNC was associated with expression levels of miR-200b-3p, miR-9, miR-483-5p, miR-22, miR-572, miR-638, miR-1234, miR-103, miR-29a, miR-let-7c, miR-196a, [58,70,71,72,73,74]. Only two miRNAs (miR-9 and miR-483-5p) have been identified, by two studies each, as being dysregulated in blood samples from HNC patients. Both of these miRNAs were described previously as being deregulated in tissue specimens [83,84] (Table 2).

Given the strong association of NPC with EBV infection, it is noteworthy that high pretreatment expression of miR-BART7-3p was shown recently to be predictive of a higher risk of distant metastasis, and thus a poor prognosis (Hazard ratio, 2.94; 95% CI, 1.44–5.98, *p* = 0.003), in a multivariate analysis [53]. Similarly, posttreatment levels of miR-BART7-3p have been found to be predictive of a poor prognosis [53].

## 6. Conclusions and Future Perspectives

Although ctDNA assessment has been incorporated in several clinical fields, including prenatal testing and cancer diagnostics, ctRNA assessment has not been developed. Progress has been limited by the number of signatures available in the literature, which for technical reasons are not amenable to direct comparison based on sensitivity, specificity, and accuracy data. The advancement of circulating ncRNA expression analysis to a point of clinical utility will require uniform standards with respect to biological sample material (e.g., serum, plasma), collection methods, downstream processing, and validation. Finally, while some information has been collected regarded the role of circulating ncRNAs in (early) diagnosis, there remains a prominent dearth of information regarding follow-up, including the identification of biomarkers of residual disease and treatment responsivity, which will require systematic longitudinal sampling prior to treatment as well as sampling immediately after treatment, and long-term posttreatment monitoring over multiple time points.

## Figures and Tables

**Table 1:** Circulating miRNAs and miRNA signatures useful for NPC ^1^ diagnosis.

miRNA	Method	Biotype	Compared Groups	Reference
BART13-3p; BART9-3p	Sequencing	Serum	NPC vs. HS ^2^	[52]
13 miRNAs	Sequencing	Plasma	NPC vs. cancer-free	[58]
8-miR	Microarray	Whole blood	NPC vs. HS	[55]
16-miR	Microarray	Whole blood	NPC vs. HNC ^3^ + HS	[55]
miR-17, miR-20a, miR-29c, miR-223	Microarray	Whole blood	NPC vs. non-cancer donors	[56]
miR-548q; miR-483-5p	Microarray	Plasma	NPC vs. CN ^4^ controls	[57]
BART7-3p; BART13-3p	RT-qPCR ^5^	Plasma	NPC vs. non-NPC (HS, CN, HNSCC ^6^)	[53]
BART2-5p	RT-qPCR	Serum	NPC vs. non-cancer	[54]

^1^ NPC, nasopharyngeal cancer; ^2^ HS, healthy subject; ^3^ HNC, head and neck cancer; ^4^ CN, chronic nasopharyngitis; ^5^ RT-PCR, reverse transcriptase quantitative polymerase chain reaction; ^6^ HNSCC, head and neck squamous cell carcinoma.

**Table 2:** Circulating miRNAs allowing prognostication of HNC ^1^.

miRNA	Method	Biotype	Type	Reference
miR-103	Sequencing	plasma	NPC ^2^	[58]
miR-29a	Sequencing	plasma	NPC	[58]
miR-let7c	Sequencing	plasma	NPC	[58]
miR-483-5p	Sequencing	plasma	NPC	[58,74]
miR-22; 572; 638; 1234	Microarray	serum	NPC	[71]
miR-196a	RT-qPCR ^3^	plasma	OSCC ^4^	[70]
miR-9	RT-qPCR	serum	OSCC	[72]
miR-200b-3p	RT-qPCR	plasma	OSCC	[73]

^1^ HNC, head and neck cancer; ^2^ NPC, nasopharyngeal cancer; ^3^ RT-qPCR, reverse transcriptase quantitative polymerase chain reaction; ^4^ OSCC, oral squamous cell carcinoma.
